# Ovarian Cancer: Opportunity for Targeted Therapy

**DOI:** 10.1155/2012/682480

**Published:** 2011-12-22

**Authors:** Tomoko Tagawa, Robert Morgan, Yun Yen, Joanne Mortimer

**Affiliations:** ^1^Department of Medical Oncology, City of Hope National Medical Center, Duarte, CA 91010, USA; ^2^Department of Medical Oncology and Therapeutics Research, City of Hope National Medical Center, 1500 E Duarte Rd, Duarte, CA 91010, USA

## Abstract

Ovarian cancer is a common cause of cancer mortality in women with limited treatment effectiveness in advanced stages. The limitation to treatment is largely the result of high rates of cancer recurrence despite chemotherapy and eventual resistance to existing chemotherapeutic agents. The objective of this paper is to review current concepts of ovarian carcinogenesis. We will review existing hypotheses of tumor origin from ovarian epithelial cells, Fallopian tube, and endometrium. We will also review the molecular pathogenesis of ovarian cancer which results in two specific pathways of carcinogenesis: (1) type I low-grade tumor and (2) type II high-grade tumor. Improved understanding of the molecular basis of ovarian carcinogenesis has opened new opportunities for targeted therapy. This paper will also review these potential therapeutic targets and will explore new agents that are currently being investigated.

## 1. Introduction

Ovarian cancer is the most common cause of gynecologic neoplasm and is the fifth cause of cancer mortality in women. The high mortality rate in women with epithelial ovarian cancer (EOC) is due to its detection at advanced stages. Even though there have been improvements in surgical techniques and treatment options, five-year survival for stage III and IV ovarian cancer still remains at approximately 45% [1].

Known risk factors of EOC include nulliparity, early menarche, late menopause, and age. A particularly significant risk factor is a strong family history of breast and ovarian cancer. 10%–15% of women with ovarian cancer have genetic predispositions of BRCA1 and BRCA 2 mutations [2]. BRCA1 is associated with a 40% lifetime risk of ovarian cancer, and BRCA 2 has an approximately 15% lifetime risk of ovarian cancer. Epidemiological studies show a reduction in the incidence of EOC in developed countries [2].

Part of the complexity of EOC lies in its heterogeneity. EOC can be classified into diverse group of tumors on the basis of morphology and molecular genetic features. This paper will review the current understanding of the molecular and morphologic heterogeneity of EOC as well as possible explanations of pathogenesis that contribute to the heterogeneity.

## 2. Tumor Origin and Pathogenesis

EOC origins are difficult to ascertain, because the majority of cases are diagnosed at late stages. Thus, there are limited records regarding early-stage disease. Historically, EOC is thought to originate from the ovarian epithelial surface and undergoes progressive dedifferentiation and spreads to the pelvic and abdominal cavities prior to metastasizing to distant organs [2, 3]. However, EOC which predominantly consists of serous, endometrioid, and mucinous cell types is morphologically columnar and ciliated, similar to Mullerian epithelial cell lining of the endometrium, endocervix, fallopian tube, and gastrointestinal tract [3]. The ovarian epithelial surface, where these cells are purported to have originated from, consists of a single mesothelial layer of cells that are flattened and squamous-like. To explain this discrepancy, the traditional theories suggest that the mesothelial lining of the ovary invaginates to form paraovarian cysts that acquire Mullerian cell lining features and undergo malignant transformation [4]. The enlarging tumor envelops the ovary and is diagnosed as an adnexal mass of ovarian origin [5, 6].

Increasing evidence now suggests that the Fallopian tube may be an alternative site of tumor origin in many diagnosed as primary EOC [5]. In older studies, the origin of EOCs were presumed to be the ovaries, and Fallopian tubes were typically not examined. However, more recently, observational studies have shown that in situ and early invasive tubal carcinomas occur in women with a genetic predisposition for ovarian cancer [5, 7, 8]. Furthermore, over 70% of nonhereditary ovarian cancer and peritoneal high-grade serous carcinomas revealed serous epithelial carcinoma in the Fallopian tube and mucosal tubal involvement [9].

The fimbria of the Fallopian tube are abundant with angiolymphatic vasculature and are in direct contact with the basement membrane of the Fallopian tube. Through this vasculature, the serous tubal intraepithelial carcinoma may conceivably disseminate to the surface of the ovary and peritoneum without invasive growth from the Fallopian tube [10, 11]. Therefore, rather than the tumor originating from a cyst that developed from the mesothelial lining of the ovary, tubal epithelium may directly implant into the surface of the ovary to form an inclusion cyst which subsequently develops into tubal epithelial carcinoma [3, 10]. An alternative possibility is that normal tubal intraepithelial cells implant into the ovary at the time of ovulation and develop into inclusion cysts that transform into carcinoma over time [4, 10].

Similarly, the endometrioid and clear cell carcinomas are thought to originate from endometriosis. According to this theory, endometrial cells “escape” the uterus via retrograde menstrual flow and implant the ovary or pelvic cavity secondarily. This mechanism has been supported by multiple morphologic and molecular studies [12, 13].

## 3. Morphologic and Molecular Characteristics

EOC was initially categorized into invasive serous carcinoma and serous borderline tumor (SBT) which was defined as a low malignant potential carcinoma lacking invasive growth. More recently, SBT was further subdivided into (1) atypical proliferative serous tumor (APST) and (2) micropapillary serous carcinoma (MPSC), a possible precursor to low-grade serous carcinoma (LGSC) [10]. Previously, serous carcinoma was thought be a spectrum of disease, where LGSC progressed to high-grade serous carcinoma (HGSC) over time. However, with the understanding that LGSC arises from SBT, high-grade invasive carcinoma is thought to be a disparate entity. This resulted in a model of ovarian carcinogenesis that consists of two distinct pathways and subtypes [3, 4, 14].

The two subtypes are low-grade (type I) and high-grade (type II). Type I tumors are typically indolent, slow growing tumors that are often detected at early stages. Type I tumors include low-grade serous, low-grade endometrioid, clear cell, and mucinous carcinomas [15, 16]. Type II includes high-grade serous, high-grade endometrioid, mixed mesodermal (carcinosarcoma), and undifferentiated carcinomas. Type II tumors are typically diagnosed in advanced stages. The majority of EOC, approximately 75%, are type II aggressive tumors [17, 18]. Type I tumors have a median survival of 81 months compared to 57 to 65 months in type II tumors.

Molecular and genetic differences are now being recognized to further understand the distinction between these two subtypes. The main genetic difference between the two subtypes that explains duality in their malignant potentials is (1) type I tumors have genetically stable and isolated mutations and (2) type II tumors have significant genetic instability and involve p53 mutations that result in a more aggressive and invasive phenotypic expression [4].

Mutational analysis and genetic expression studies have shown that APST, MPSC, and LGSC share molecular mutations that are significantly different from molecular alterations in HGSC [10]. Type I tumors typically involve mutations in a number of genes such as KRAS, BRAF, PTEN, PIK3-CA, and CTNNB1 which encodes beta-catenin [10, 14]. Mutations in these upstream regulators result in constitutive activation of the MAPK signaling pathway. Up to 70% of MPSC and LGSC have been shown to express active MAPK signaling [4, 15]. Her2/neu mutations have been detected in approximately 10% of Type I tumors and appear to be mutually exclusive with having KRAS and BRAF mutations [10, 15]. Type I tumors rarely express p53 mutations. Furthermore, to further explain the phenotypic association between LGSC and MPSC that are not shared with APST, MPSC is more molecularly similar to LGSC than APST. On the basis of mutational analysis of clear cell carcinomas, the PI3K/PTEN pathway appears to be the most commonly deregulated [19]. While PTEN mutations are present in low-grade endometrioid histologies, they also have alterations in the Wnt/*β*-catenin pathways and CTNNB1 mutations [20]. Therefore, among the type I tumor subtypes, variable genetic alterations have been identified that explain their phenotypic differences.

In contrast, type II tumors have TP 53 mutations in up to 95% of the cases. They are also characterized by genetic instability and high frequencies of DNA copy number gains and losses. They rarely contain KRAS and BRAF mutations. A recent publication in Nature identified 9 significantly mutated genes in high-grade serous ovarian cancer. The most common were RB1 mutations (67%), TP 53 mutations (96%), PI3/Ras pathways (47%), and BRCA 1 or 2 mutations (22%) [21]. De novo, nonfamilial cases of BRCA1 and BRCA2 inactivation are often associated with hypermethylation. In a genomic analysis of high-grade ovarian cancer, 11% of BRCA1 silencing was the result of hypermethylation and epigenetic modification rather than mutations. Studies have shown that mutated or hypermethylated BRCA carriers respond to PARP inhibitor therapy [22, 23].

## 4. MicroRNA

MicroRNAs are short nucleotide sequences that are noncoding RNAs that are involved in the regulation of posttranscriptional genes. MicroRNAs are critical in cell development but may also contribute to tumor origin [24–27]. MicroRNAs have been found to be differentially expressed between ovarian carcinoma compared to normal ovarian epithelial cells [28]. Upregulation or downregulation of microRNA that regulate oncogenes and tumor suppressor genes, respectively, can induce malignant transformation. Dysregulation of microRNA expression has been associated with high-grade serous ovarian carcinoma. The relationship between such microRNA dysregulation and BRCA 1 or 2 expression is being explored [28]. Furthermore, microRNA have previously been found to regulate cellular differentiation [29, 30] and may play a role in epithelial to mesenchymal cell transformation during carcinogenesis of ovarian epithelial cells. Exploration of microRNA involvement in ovarian carcinogenesis has recently focused attention on Let-7 microRNA and HMGA2 gene regulation.

## 5. HMGA2 and Let-7

HMGA2, a high-mobility-group AT-hook protein, is a nonhistone DNA-binding factor that attaches to AT-rich sequences in the minor groove of the DNA helix. It is an important regulator of cell growth, differentiation, apoptosis, and malignant transformation. HMGA2 expression is regulated by the microRNA Let-7. Preclinical studies have evaluated the role of let-7 as a tumor suppressor gene to the oncogenic mechanism of HMGA2 [31]. When the let-7 activity is downregulated, HMGA2 activity is no longer repressed and contributes to malignant transformation [31]. HMGA2 overexpression has been identified in 65% of ovarian carcinoma but are rarely expressed in normal ovarian epithelial cells [32–34]. Furthermore, HMGA2 overexpression has been associated with high-grade serous ovarian carcinoma [32, 33]. The Let-7 HMGA2 dysregulation may be a key factor in ovarian carcinogenesis distinguishing low-grade or type I from high-grade or type II EOC [35].

In our institution, we compared the HMGA2 expression in serous OC and endometrioid OC surgical specimens. In this study, we attempted to distinguish expression patterns on the basis of the phenotypic grade of the OC. Fourteen consecutive endometrioid OC were analyzed and twelve consecutive high-grade serous carcinomas were analyzed for HMGA2 expression by immunohistochemical staining. High-grade serous carcinoma was associated with greater HMGA2 expression compared to endometrioid OC. Longitudinal evaluation is pending to evaluate the prognostic significance of HMGA2 expression and clinical outcome [41].

Other studies have further evaluated the correlation of HMGA2 overexpression and p-53 mutations [32] further demonstrating the tumorigenesis of type II high-grade EOC that is distinct from type I low-grade EOC. Furthermore, animal models have shown that HMGA2 silencing is associated with reduction in tumor growth and increased apoptosis of tumor cells [34]. This suggests that HMGA2 may be a possible target in ovarian cancer therapy.

## 6. Opportunities for Targeted Therapy

Despite optimal surgical and cytotoxic treatment of advanced ovarian cancer, only 10% to 15% achieve long-term remission, and the majority will face recurrent disease [42, 43]. While there is a role for chemotherapy in recurrent disease, the effects are often short lived due to development of chemoresistance [44]. Targeted therapies in ovarian cancer are currently being investigated to find novel ways to overcome chemoresistance. These molecular targets of therapy include: VEGF (antiangiogenesis), EGFR tyrosine kinase, HER2 receptor, PARP, and MAPK/BRAF/MEK pathways (See Table 1).

## 7. Antiangiogenesis

Angiogenesis has been shown to be a key component in ovarian cancer metastasis and ascites development [45]. Vascular endothelial growth factor (VEGF) is a critical regulator of angiogenesis and is involved in various aspects of ovarian carcinogenesis [46]. Antiangiogenic therapy has been shown to have activity in ovarian cancer. Bevacizumab is a monoclonal antibody that targets VEGF-A. Two phase II trials with single-agent bevacizumab have shown 16% to 21% response [47, 48]. Phase III studies involving chemotherapy with or without bevacizumab presented during the 2011 Annual Meeting of the American Society of Clinical Oncology (ASCO) showed a trend toward an overall survival benefit in patients treated with bevacizumab in addition to chemotherapy in the first line setting with HR = 0.64  *P* = 0.0022 in those with poor prognosis disease (ICON7). Similar data were reported in patients treated for platinum sensitive recurrent ovarian cancer with median overall survival of 35.5 months (+bevacizumab) versus 29.9 months (−bevacizumab) and HR = 0.751  *P* = 0.094 (OCEANS) [49].

Mammalian target of rapamycin (mTOR) is involved in cell growth and proliferation and induces increases in VEGF and platelet derived growth factor (PDGF) that ultimately activate angiogenesis. mTOR inhibitors have also shown single agent activity in the treatment of clear cell ovarian carcinoma [50]. The antiangiogenic effects are thought to be synergistic with that of bevacizumab. Results of a phase II study combining the mTOR inhibitor, temsirolimus, with bevacizumab suggest possible synergy [49]. Other phase I and II trials involving antiangiogenic therapy are showing activity as single agents or synergistically with chemotherapy. These agents include (1) VEGF trap (Aflibercept) which is a fusion protein that acts as a high-affinity VEGF receptor blocker, (2) Sunitinib (a PDGF and VEGF receptor inhibitor), (3) Vatalanib (a pan-VEGF receptor inhibitor), (4) Motesanib (a multikinase inhibitor of PDGF, VEGF, and cKIT), and (5) Cedarinib (a VEGF tyrosine kinase receptor inhibitor).

## 8. Anti-HER2 Therapy

Her2 expression in ovarian cancer has been variable. Many studies evaluate HER2 expression in ovarian cancer as a positive or negative expression and do not describe the staining intensity to characterize the overexpression of HER2 [51]. The studies that have evaluated HER2 overexpression demonstrate significant variability in intense HER2 staining from 1.8% to 35% [43, 51–53]. Studies have suggested that gene amplification does not always correlate with HER2 protein overexpression [54]. Furthermore, some data suggest that HER2 overexpression is mostly found in high-grade serous histology as opposed to low-grade endometrioid [55]. Her2 positive expression has demonstrated association with survival in patients with EOC [53, 56–58]. More recently, HER2 gene status was evaluated from the GINECO study, and no survival differences were detected in patients with or without HER2 overexpression who were treated with carboplatin/paclitaxel [59]. This suggests an association between HER2 status and paclitaxel sensitivity.

As a result of such variability in HER2 expression in ovarian cancer, the role of anti-HER2 therapy is unclear. Anti-HER2 therapy with trastuzumab and pertuzumab have shown modest activity in ovarian cancer [40, 60]. Preclinical studies initially suggested activity in tumors without HER2 overexpression [61]. However, subset analyses of phase II trials indicate that pertuzumab has better activity in those with HER2 overexpression [60]. HER2 expression in EOC has not been studied as extensively as in breast cancer, and there are many inconsistent data. Thus, the limitations to anti-HER2 therapy hinges on further careful examination of HER2 oncogene as a potential prognostic, predictive, and therapeutic target.

## 9. EGFR Tyrosine Kinase Inhibitors

EGFR tyrosine kinase inhibitors are also being explored including cetuximab, lapatinib, and erlotinib [62, 63]. A phase II study of erlotinib with carboplatin has shown activity in platinum sensitive recurrent ovarian cancer (57% objective response rate in platinum sensitive and 7% in platinum-resistant patients) [64]. A phase II study with erlotinib, carboplatin, and paclitaxel in the first line setting showed no statistically significant pathologic complete response rates compared to historical controls [65]. A Phase I/II study of lapatinib with carboplatin and paclitaxel in recurrent stage III or IV ovarian and breast cancer proved safe with favorable response rates [66]. Recently, a phase II study was published which explored lapatinib combined with topotecan in platinum refractory or resistant ovarian cancer. This study showed minimal clinical activity with significant hematologic grade 3 and 4 toxicities [39]. A Phase II study with cetuximab and carboplatin in EGFR-positive ovarian cancer showed modest activity where 9 of 28 patients achieved objective response and 8 of 28 patients had stable disease [67]. Toxicities in this study included acneiform rash and hypersensitivity reactions. Another phase II study with cetuximab, carboplatin, and paclitaxel failed to demonstrate progression-free survival benefit compared to historical data [62].

## 10. PARP Inhibitors

Poly (ADP-ribose) polymerase (PARP) inhibitors block base excision repair. For tumors that lack DNA repair mechanisms due to BRCA1/BRCA2, HNPCC, Fanconi Anemia, and other genetic mutations, inhibiting alternate repair pathways with PARP inhibition may increase antitumor selectivity and improve chemotherapy sensitivity. Three phase II studies with PARP inhibitors, olaparib and iniparib, show activity in recurrent platinum sensitive ovarian cancer in combination with chemotherapy or as single-agent maintenance after chemotherapy [49]. Olaparib as a maintenance therapy has also been shown to improve progression-free survival by 3.6 months compared to placebo in platinum-sensitive relapsed serous ovarian cancer. (ASCO 2011 abstract 5003) There is currently an ongoing NCI sponsored randomized Phase II study that explores ABT 888 (veliparib) with cyclophosphamide in BRCA positive ovarian and triple negative breast cancer [68].

## 11. Epigenetic Studies

The pathophysiology of cancer is not only the result of inherited or sporadic genetic mutations but is also the result of epigenetic modifications in the genome. Histone hypoacetylation and abnormal DNA methylation also contribute to tumorigenesis and chemotherapy resistance. A phase II study with the DNA hypomethylating agent, decitabine, showed improved response rates in platinum-resistant ovarian cancer when added to carboplatin therapy at low dosages, where 9 out of 17 patients had progression-free survival at 6 months (ASCO 2011 abstract 5011) [49]. Other studies with the histone deacetylase inhibitor, belinostat, and the proteosome inhibitor, carfilzomib, are being explored in ongoing phase II trials.

## 12. MAPK/BRAF/MEK Pathway

One of the major pathways that regulate cellular growth is extracellular-related kinase (ERK) which triggers a cell surface-receptor mediated signaling cascade involving Ras, Raf, MEK [mitogen-activated protein (MAP)/ERK kinases] and ERK. As described previously, BRAF mutations are commonly associated with low-grade ovarian carcinoma. BRAF mutations result in the constitutive activation of the MAP kinase/MEK pathways. Furthermore, preclinical models have shown that in addition to BRAF mutations in low-grade tumors, Raf-1 isoform predominantly mediates ovarian cancer cell growth compared with Raf-A or B-Raf isoforms [69]. There have also been reports demonstrating reduced survival in ovarian cancer patients with increased expression of Raf-1 [70]. Thus, the MAPK/BRAF/MEK pathway can be a target in both high-grade and low-grade ovarian cancer. MET tyrosine kinase cell surface receptor is involved in RAF and MAP kinase activation pathways, and its inhibition results in downstream suppression of RAF and MAP kinase activity. The potent MET inhibitor, cabozantinib, showed activity in advanced ovarian cancer irrespective of platinum sensitivity [49].

## 13. Let-7 MicroRNA Therapy

Among the emerging next-generation therapies are the microRNA therapeutics. Preclinical mouse models with exogenous Let-7 microRNA have shown suppression of cell proliferation in breast cancer cells [71, 72]. There are significant limitations to clinical applicability of microRNA technology at this time due to a limited understanding of the Let-7 mechanism and with methods of delivery.

## 14. Conclusion

Epithelial ovarian carcinoma is a highly heterogeneous disease process that is associated with significant mortality and morbidity. Recent progress in molecular characteristics of ovarian cancer has helped delineate the origins of carcinogenesis, particularly, a model of tumorigenesis which is based on a dichotomous theory of (1) low-grade or type I ovarian cancer associated with gene stability and multiple isolated mutations and (2) high-grade type II ovarian cancer associated with genetic instability and p53 mutations. Continued evaluation of the molecular makeup of ovarian carcinoma is critical in the further identification of treatment targets and improved clinical outcome. 

## Figures and Tables

**Table 1:** Potential targets of therapy in ovarian cancer.

Targets	Inhibitors	Studies	NCI study
			GOG 218
VEGF	Bevacizumab	Phase III [49]	ICON 7
			GOG 213
			OCEANS

VEGF TKI	Sunitinib	Phase II	NCT00979992
			NCT00768144
			NCT00388037
		
	Vatalanib	Phase I (+doce)	NCT00268918
		
	Sorafenib	Phase II (+carb/pacli)	NCT00390611
		Phase II (+topo)	NCT01047891
		Phase II	NCT00522301
		Phase II (+bev)	NCT00436215
		Phase II [36]	NCT00093626
		Phase II (+carb/pacli)	NCT00096200
		
	Vandetanib	Phae I/II (+bortezomib)	NCT00923247
		Phase II	NCT00445549
		Phase II(+/− doce)	NCT00872989
		
	Cediranib	Phase I/II(+olaparib)	NCT01116648
		Phase II	NCT00275028
		Phase II (+temsirolimus)	NCT01065662
		
	Pazopanib	Phase I/II (+cyclophosphamide)	NCT01238770
		Phase II	NCT01262014
		Phase I/II [37]	NCT01035658
		Phase II	NCT00281632
		Phase III	NCT00866697
		
	Vargatef	Phase I (+everolimus)	Pending
		
	AMG 706	Phase II	NCT00574951

VEGF TRAP	Aflibercept	Phase II	NCT00327171
		Phase II (+doce)	NCT00436501

PI3K-PTEN-Akt-mTOR pathway	Temsirolimus	Phase I (+lip Doxo)	NCT00982631
		Phase II	NCT00926107
		Phase II (carb/pacli)	NCT01196429
		Phase II	NCT00429793
		Phase I/II(+bev)	NCT01010126
		
	Everolimus	Phase II (+bev)	NCT01031381
		Phase II (+/− bev)	NCT00886691
		Phase II (+lip doxo)	NCT01281514
		
	Ridaforolimus	Phase I (+carb/pacli)	NCT01256268

EGFR	Cetuximab	Phase II (carb/pacli)	NCT00063401/
		Phase II (+carb)	NCT00086892
		
	Erlotinib	Phase II (+bev) [38]	NCT00130520
		Phase II (+topo)	NCT01003938
		Phase I/II (+carb/pacli)	NCT00217529
		Phase II (+carb)	NCT00030446
		Phase II (+bev after carb/pacli/bev)	NCT00520013
		
	Lapatinib	Phase II (+topo) [39]	NCT00436644
		Phase II	NCT00113373
		Phase I/II (+carb/pacli)	NCT00316407

HER2	Trastuzumab	Phase II [40]	
		
	Pertuzumab	Phase II	NCT00058552
		Phase II (+gemcitabine)	NCT00096993
		
	Lapatinib	See above	

PARP	ABT 888	Phase II (+temoz versus lip doxo)	NCT01113957
		Phase II (+cyclophosphamide)	NCT01306032
		Phase I/II (+topo)	NCT01012817
		Phase I (+carb/pacli/bev)	NCT00989651
		
	Olaparib	Phase II (+carb/pacli)	NCT01081951
		Phase I (+carb)	NCT00647062
		Phase II	NCT00679783
		Phase II	NCT00494442
		Phase II (versus lip doxo)	NCT00628251
		Phase II	NCT00753545
		Phase II	NCT01033123
		
	Iniparib*	Phase II (carb/gemcitabine)	NCT01033292
		Phase II	NCT00677079

Epigenetic	Decitabine	Phase II (+carb)	NCT00477386
		Phase I (+doxorubicin/vaccine)	NCT00887796
		
	Belinostat	Phase I/II (+carb or pacli)	NCT00421889

MAPK/RAF/MEK pathway	Cabozantinib	Phase I	NCT00940225

HMGA2	Let-7 microRNA	Preclinical	

*Iniparib as a true PARP inhibitor is currently under investigation.

Doce: docetaxel, Carb: carboplatin, pacli: paclitaxel, topo: topotecan, bev: bevacizumab, temoz: temozolomide, and lip doxo: liposomal doxorubicin.
